# 38189 Potential effect of serum from hypertensive donors on PP2A expression and activity in endothelial cells

**DOI:** 10.1017/cts.2021.434

**Published:** 2021-03-30

**Authors:** Dulce H. Gomez, Maitha Aldokhayyil, Austin T. Robinson, Michael D. Brown

**Affiliations:** 1 Auburn University; 2 Imam Abdulrahman Bin Faisal University

## Abstract

ABSTRACT IMPACT: Racial differences in the prevalence of hypertension and endothelial (dys)function are well established, yet research investigating the mechanism(s) underlying this disparity is still lacking. OBJECTIVES/GOALS: Investigate the influence of race and the effect of serum collected from hypertensive donors on Protein Phosphatase 2A (PP2A) and endothelial nitric oxide synthase (eNOS) expression and activity in human umbilical vein endothelial cells (HUVECs) from Caucasian (CA) and African American (AA) donors. METHODS/STUDY POPULATION: HUVECs from 3 CA & 3 AA donors were cultured in parallel. Experiments were conducted between passages 5-7. At ?90% confluency, cells were serum starved ˜12hrs prior to incubating for 24 or 48 hours in one of the following conditions: 1) Control (Fetal Bovine Serum), 2) serum from normotensives (NT; 5 CA & 5 AA donors), or 3) serum from hypertensives (HT; 5 CA & 5 AA donors). NT and HT serum was pooled from donors with the following characteristics: Male, 30-50 years, nonsmokers, no comorbidities, and non-obese (BMI < 30 kg/m2). Western blotting was used to measure protein expression of total eNOS, p-eNOSS1177, total PP2A, and p-PP2AY307. For activity p-eNOSS1177/total eNOS and p-PP2AY307/ total PP2A ratio was used. A two-way ANOVA was used for statistical analysis. RESULTS/ANTICIPATED RESULTS: Irrespective of the donors’ race, there was no influence of serum treatment or interaction effect in any of the measured proteins of interest. Moreover, compared to CA, HUVECs from AA had lower expression of eNOS irrespective of condition (race p=0.01). Compared to CA, HUVECs from AA tended to have lower expression of p-eNOSS1177 irrespective of condition (race p=0.07). However, there was no racial differences in eNOS activity (p=0.68). There was no racial difference in the expression of PP2A (p=0.35), p-PP2AY307 (p=0.30), or PP2A activity (p=0.97) in all conditions. DISCUSSION/SIGNIFICANCE OF FINDINGS: Our preliminary results suggest no influence serum constituents from hypertensive donors or race on PP2A or eNOS expression and activity in HUVECS. Future research should consider conducting proteomics profiling to compare NT and HT serum.

